# Repositioning and stabilization of the radial styloid process in comminuted fractures of the distal radius using a single approach: the radio-volar double plating technique

**DOI:** 10.1186/1749-799X-5-55

**Published:** 2010-08-11

**Authors:** Matthias Jacobi, Peter Wahl, Georges Kohut

**Affiliations:** 1 Department of Orthopaedic Surgery, Hôpital Cantonal Fribourg, 1708 Fribourg, Switzerland

## Abstract

**Background:**

A possible difficulty in intra-articular fracture of the distal radius is the displacement tendency of the radial styloid process due to the tension of the brachioradialis tendon.

**Methods:**

Ten patients treated within one year for complex distal radius fractures by double-plating technique with a radial buttress plate and volar locking plate, through a single volar approach, were followed prospectively during 24 months. Outcome measures included radiographic follow-up, range of motion, grip strength and score follow-up (VAS, Gartland-Werley score and patient-rated wrist evaluation).

**Results:**

Ten patients with intraarticular distal radius fractures with dislocation of the radial styloid process were treated with this technique. This resulted after 24 months in good clinical outcome (mean visual analog scale 0.9; almost symmetric range of motion; mean Gartland-Werley score 2 ± 3; mean patient-rated wrist evaluation 3.2 ± 2.4). Radiologic evaluation according to the Dresdner Score revealed anatomic reduction without secondary dislocation during the follow-up and uneventful consolidation.

**Conclusions:**

The described technique strongly facilitates anatomic reduction and stable fixation of intra-articular distal radius fractures with dislocation of the radial styloid process and leads to satisfactory clinical and radiographic outcome.

## Background

During the last decade, open reduction-internal fixation (ORIF) has become increasingly popular, and is used more frequently for distal radius fractures [1-7]. It provides some advantages over external percutaneous fixation techniques. The functional (and therefore faster) rehabilitation is advantageous; it allows for an earlier return to work and less wound care is needed. Nevertheless, final outcome is similar [8,9].

There has been a tendency during the last five years for ORIF to be done predominantly with volar implants and angular stability [10,11]. With this technique, most fracture types can be treated with good-to-excellent results. Functional outcome is similar to results obtained by a dorsal approach, avoiding tendon irritation [6,10,12]. Implant removal is therefore dispensable in many cases.

**Figure 1:**
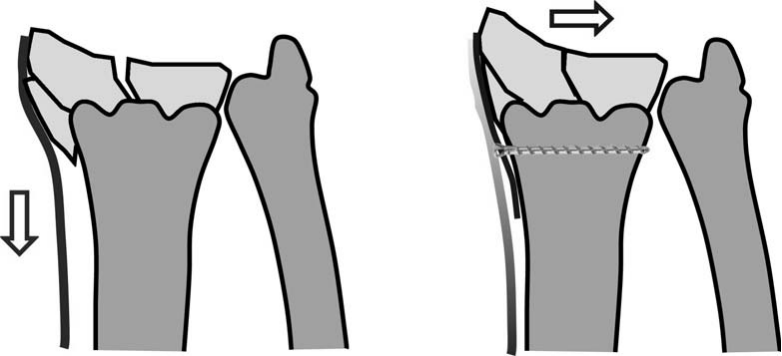
**Radial styloid dislocation and reduction**. Dislocation of the radial styloid process is favored by the traction of the brachioradialis tendon. The radial buttress plate reduces displacement and neutralizes brachioradialis traction.

A possible difficulty during reduction of distal radius fractures can be the proximal displacement and radial shift of the radial styloid process, due to the tension of the brachioradialis tendon (figure 1). This problem is often encountered in comminuted, intra-articular, and/or osteoporotic fractures. Due to patient positioning (i.e., supine position with the arm abducted; elbow extended with the forearm supinated), tension in the brachioradialis tendon is increased, which in turn worsens dislocation. Tendon release (as suggested by Orbay), direct and indirect manipulation, ligamentotaxis, the Willenegger or Kapandji technique and a change in arm position (to flexion of the elbow and pronation of the forearm) may ease repositioning [5,13,14]. Nevertheless, this can be insufficient to reduce the styloid process to an anatomic position. The three-column concept of the distal radius as proposed by Rikli respects radial dislocation of the styloid process and fixes the radial column with a separate plate [15,16]. A similar principle can also be used with volar fixation. One main difference is that the radial plate in the Riklis technique works in the sagittal plane and is not only a buttress plate, but also fixes the fracture.

In this report on single-approach radio-volar plating, we describe a simple technique to overcome the dislocation tendency of the radial styloid process and ease anatomic reduction. We detail this surgical technique with clinical and radiological outcome of treated patients.

## Methods

A consecutive series of patients treated with radio-volar double plate fixation (radial buttress plate and volar locked plate) for fractures of the distal radius between 1 January and 31 December 2006 at our institution were selected and controlled prospectively for this study. Patients requiring supplementary fixation (e.g., dorsal plating or a supplementary approach such as a dorsal approach) where excluded.

Indication for volar and, if judged necessary, radial plating, was a choice of the surgeon on call based on standard recommendations in our department. A radial plate was used only in cases of difficult repositioning with radial shift and proximal displacement of the styloid process if satisfactory reduction was not achieved otherwise (see background). The decision to add a radial plate was therefore taken intraoperatively.

Preoperatively, plain radiographs were taken and in six cases CT was done (one patient was too obese, and in three cases the surgeon considered it to be unnecessary). Surgery was carried out immediately or once soft-tissue swelling was acceptably low.

### Surgical technique

Patients were placed on the operating table in the supine position with abduction of the arm and supination of the forearm. A tourniquet was used, and standard disinfection and draping carried out. A distal Henry approach was carried out in the interval between the flexor carpi radialis tendon and the radial artery. The distal part of the pronator quadratus muscle was released from the radius. Care was taken to release only the amount of muscle necessary for fracture exposure and plate insertion. In most cases, the proximal part of the plate was placed under the main body of the pronator quadratus muscle. The brachioradialis tendon was partially released from the radial aspect of the styloid; fibers that insert proximally to the fracture remained intact, but most of the fibers that insert onto the styloid fragment were sectioned, without Z-lengthening. We assumed that the remaining insertion of the brachioradialis would be sufficient to hold this tendon in an appropriate place during fracture healing. If needed, the sheath of the abductor longus and extensor brevis tendons was sectioned, but care was taken to avoid direct contact between these tendons and the plate on the radial side. Satisfactory anatomic repositioning and/or stabilization of the radial styloid process was not possible in all patients, so a radial plate, through the same approach, was added. An AO 2.7-mm 1/3-tube plate (Synthes^®^, Oberdorf, Switzerland) or a straight Aptus Radius plate, 2.5 mm (Medartis^®^, Basel, Switzerland) was used in all cases. The plate was fixed as a buttress plate with one or two screws in the radial aspect of the radial shaft, just proximal to the fracture line. With this, repositioning of the fracture in the frontal plane was achieved (figure 1). The main volar plate (Aptus Radius, 2.5 mm; Medartis^®^) was added after definitive repositioning of the fracture in the sagittal plane. Screws with angular stability were used with this plate; we tried to place at least 2-3 screws in the radial styloid process. After fluoroscopic control, closure was by adaptation of the pronator quadratus muscle with absorbable sutures, and suture of the skin with non-resorbable sutures.

### Rehabilitation

Active motion of fingers, elbow and forearm in pronation and supination without weight bearing was started 2-3 days after surgery for all patients. The wrist was supported on a removable splint for 6 weeks, but gentle range-of-motion exercises for flexion and extension were initiated. Weight bearing was allowed after 6 weeks if there was radiographic evidence of fracture healing.

### Outcome evaluations

Clinical and radiographic outcome evaluations were done at 6 weeks, 3, 6, 12 and 24 months after surgery. Range of motion was noted. Grip strength was measured with JAMAR hand dynamometer (JAMAR TEC, Clifton, New Jersey). Pain was evaluated with the visual analog scale [17]. Additionally all patients completed the Gartland and Werley score [18] and the patient-rated wrist evaluation [19].

**Figure 2:**
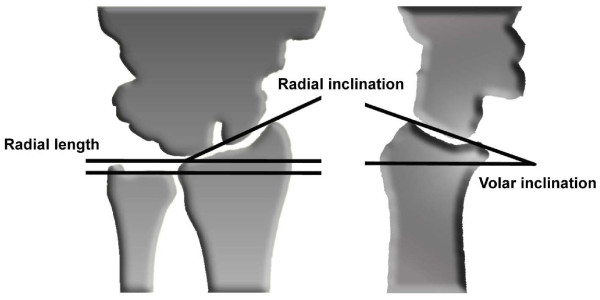
**Radiologic evaluation**. Radiologic evaluation according to the Dresdner Score included (i) volar inclination; (ii) radial inclination; (iii) loss of radial length and (iv) intraarticular steps.

Radiological analysis included fracture AO-classification. Preoperatively and postoperatively, joint inclination in the lateral view, radial inclination in the anteroposterior view, loss of radial length, as well as intra-articular steps were evaluated according to the Dresdner Score (figure 2, table 1) [20]. Time until consolidation was determined evaluating callus formation, gap-filling and restoration of bone architecture.

**Table 1:** Radiologic evaluation according to the Dresdner Score

Parameter	Rating	Points	Preoperative (n)	Postoperative (n)
Volar inclination	Norm 5-15°	0	-	8

	Deviation 5-10°	1	2	2

	Deviation >10°	2	8	-

Loss of radial length	Till 2 mm	0	-	10

	3-4 mm	1	2	-

	> 4 mm	2	8	-

Radial inclination	20-25°	0	-	6

	Deviation 5-10°	1	5	4

	Deviation >10°	2	5	-

Joint line	No steps	0	-	8

	Steps till 2 mm	1	2	1

	Steps >2 mm	2	8	1

**Mean total points preoperative**		**6.9**		

**Mean total points postoperative**		**0.9**		

### Statistical Analysis

Results were expressed as arithmetic mean (standard error of mean/and range). Calculations were performed using SPSS 15.0 LEAD Technologies, Inc..

## Results

### Baseline data

During the study period, 104 distal radius fractures were surgically treated at our institution. Of these, 53 were treated with a volar plate and, of these, 10 received an additional radial buttress plate and were therefore included in the study. Five patients were female and five were male; four left- and six right-sided fractures were involved. The mean age was 54 years (range, 20-82 years). Four patients were aged ≤36 years (high-energy trauma) and six subjects were ≥55 years (low-energy trauma with osteoporosis). All but one (23C2) were AO type 23C3 fractures. Four fractures had a volar tilt, and six had a dorsal tilt. All patients were available for complete follow-up. All patients underwent surgery by three surgeons experienced in treating patients who had undergone orthopaedic trauma.

### Clinical data

The mean visual analog scale at 24 months was 0.9. Range of motion was: flexion 39° (± 14.6/range 15-60); extension 49° (± 8.1/range 10-60) pronation: 75° (± 8.3/range 60-90); and supination: 75° (± 8.1/range 65-90). The mean Gartland and Werley score at 24-month follow-up was 2 (± 3/range 0-10) in which eight patients were rated as "excellent", one as "good" and one as "fair". The mean patient-rated wrist evaluation was 3.2 (± 2.4/range 0-7) at 24-month follow-up. Grip strength was 90% (± 9/range 80-100) of the opposite side with seven dominant and three non-dominant wrists involved. The available clinical data between 6 months and 24 months were virtually unchanged. Five of the patients had slight DeQuervain's tendonitis-like symptoms caused by the radial plate, and benefited from implant removal.

### Radiological data

The initial sagital tilt for the four volar-tilted fractures was 20° (± 12/range 4-30), and -26.5°(± 6.7/range -20 to -60) for the six dorsal tilted fractures, and was equal postoperatively and at 24 months for both groups 7.5°(± 3.5/range 0-12). Preoperative loss of radial length was 6.3 mm (± 3.7/range 2-12) and was equal postoperatively and at 24 months (0.1 mm (± 0.8/range 1 to -2)). Preoperative radial inclination was 10°(± 7.2/range -6-22) and was equal postoperatively and at 24 months (± 18.5°(4/range 15-26)). Results according to the Dresdner score are presented in table 1. Secondary dislocation between the postoperative position and the 24-month control was not observed in any patient. All fractures were partially consolidated after six weeks and completely consolidated after three months. One patient had evidence of osteoarthritic development on the 1- and 2-year radiograph.

### Illustrated case

**Figure 3:**
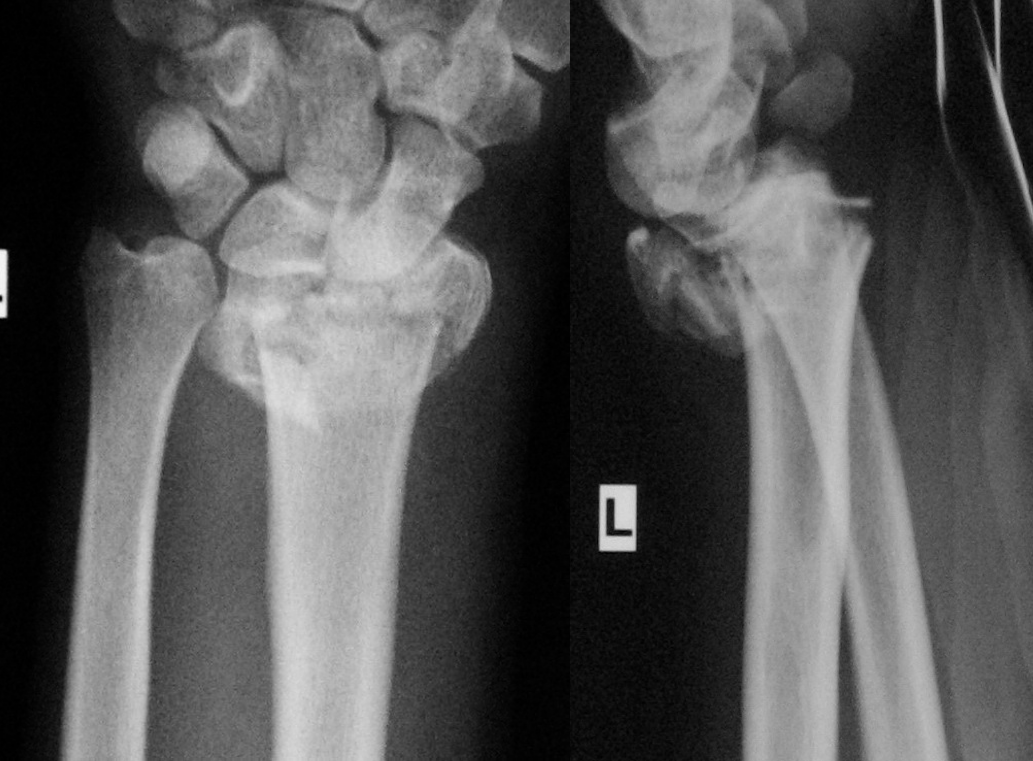
**Illustrated case (preoperative radiographs)**. Complex multifragmentary fracture of the distal radius with dorsal tilt.

**Figure 4:**
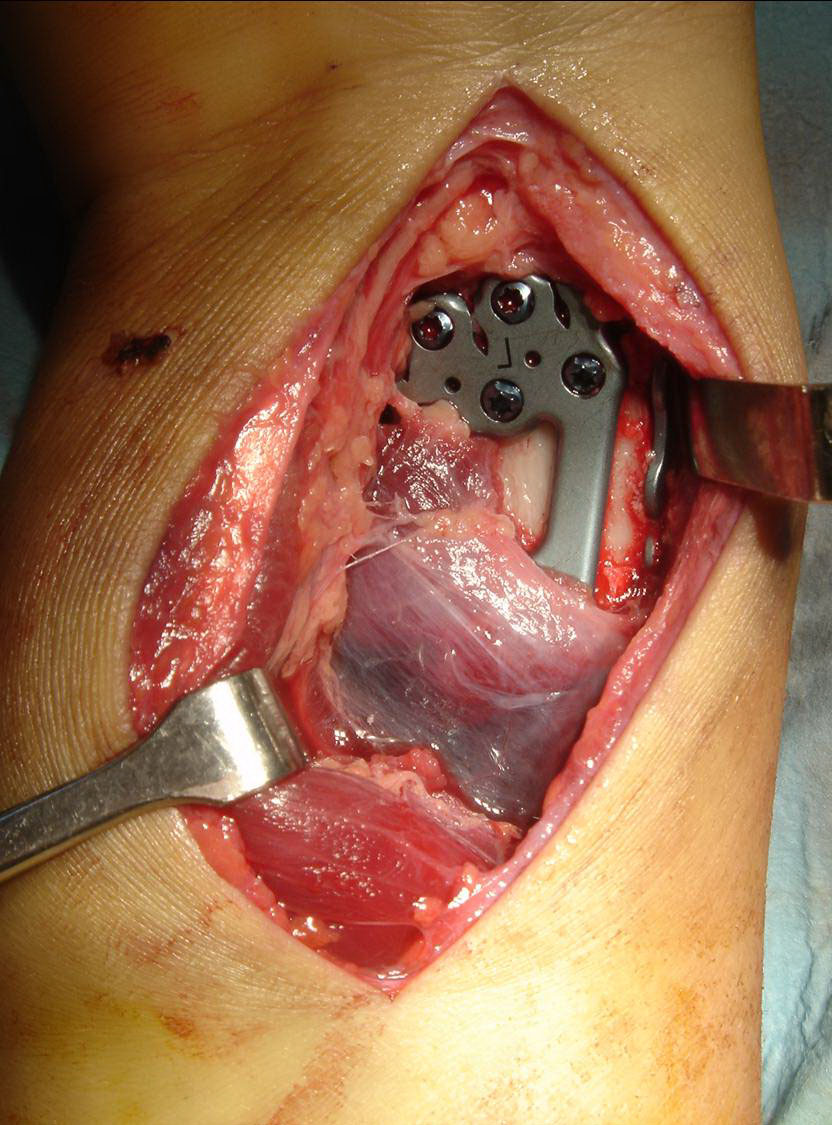
**Illustrated case (surgery)**. Intraoperative image with partially detached pronator quadratus muscle over the volar plate. The radial plate is visible on the radial border of the radius. (the image has been flipped horizontally for better comparison with the X-ray).

**Figure 5:**
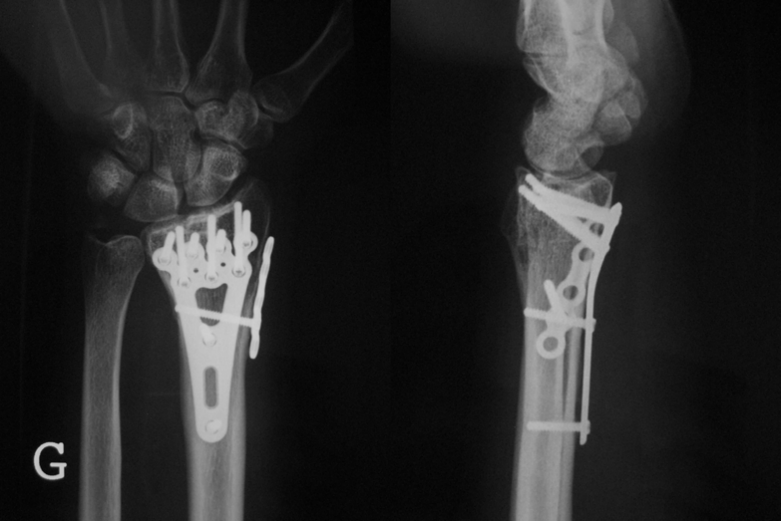
**Illustrated case (postoperative radiographs)**. The fracture is anatomically reduced and stabilized.

A 20-year-old male fell from about five meters onto his left upper extremity. Radiographic evaluation demonstrated an AO type-C3 multifragmentary dorsally tilted distal radius fracture (figure 3). He underwent surgery as described with a volar-radial double plate (figures 4 and 5). At two-year follow-up, he showed excellent outcome without pain, with free function, but with slightly reduced mobility (flexion/extension 50/0/60° (60/0/70°)). Implant removal was not necessary.

## Discussion

The presented double plating technique, with the radial plate used as a buttress plate, is a very useful tool to reduce a displaced radial styloid fragment in the frontal plane, particularly in osteoporotic bone or if the styloid fracture is multifragmentary. In these cases, anatomic reduction without the support of the radial plate can be difficult. Secondary dislocation did not occur in any of our patients. Biomechanical data for this fixation are not available, but it seems that sufficient stability was present to allow a functional rehabilitation protocol, even in osteoporotic fractures. The mechanical strength of this double-plating technique is mainly provided by the volar plate, whereas the radial plate acts as a buttress plate holding the radial styloid fragments in place. In general, only 1-2 proximal screws were used on the radial plate for this purpose.

In the present study, flexion and extension fractures are included, but they are two separate entities. However, the tendency for radial dislocation can be a common factor in both fracture types, and the radial plate can be helpful in both types.

Functional outcome for our study population reflects what is reported in the literature for distal radius fractures (although less difficult fractures were involved in most reports) [3,6,10,21]. In the follow-up, the radial plate can cause some irritation to the first extensor compartment, which is why metal removal was done in five cases. In our series, standard plates were used as the radial plate. A specially designed radial buttress plate might reduce tendon irritation. In our country, the barrier to metal removal is low, and many patients request it due to minimal symptoms. However the possibility of removal of the radial plate should be considered before wound closure in selected cases with a sufficiently stabilized radial styloid process. This is favoured if the radial styloid process fragment is not a multifragmentary fracture or osteoporotic.

The surgical approach is important. Most surgeons utilise the Henry approach to the volar side of the distal radius, entering between the flexor carpi radialis tendon and the radial artery [22]. This approach has been modified by Orbay to include release of the distal osseous insertion of the brachioradialis tendon [4]. This permits better manipulation of the fracture fragments because the brachioradialis tendon is known to be an important deforming force in such injuries [23].

If visual control of the articular surface during reduction is necessary, a single volar approach is contraindicated unless sufficient control of the articular surface can be provided by arthroscopy [24]. This can be the case in joint depression fractures, in fractures with completely displaced joint fragments or in fractures of the dorsal rim. These fractures are therefore also inappropriate for the described technique. In the present study, polyaxial locking plates were exclusively used. The technique is also suitable for uniaxial plates. This may be an advantage for stability, but is disadvantageous for optimal placement of screws.

The present study had limitations. First, radiographic evaluations were done on standard X-rays, and only in 6 of 10 cases was an initial CT available. Second, the study population was relatively small because the number of patients reflected only about 10% of the radius fractures treated surgically at our hospital. Third, a follow-up period of two years is too short to draw definitive conclusions on osteoarthritic development.

## Conclusions

The presented single-approach double plating technique with a radial buttress plate for multifragmentary distal radius fractures is useful because it facilitates anatomic repositioning and stable fixation. It is indicated only in a subgroup of patients with comminuted distal radius fractures in which displacement of the radial styloid process is difficult to manage. It leads to good clinical and radiological outcomes, as supported by our results.

## Competing interests

There was no personal or institutional financial support in relation to this study.

## Authors' contributions

MJ designed the study, collected data, prepared the artwork, drafted the manuscript and performed the data analysis. PW participated in the design of the study, participated in data collection and drafting of the manuscript. GK participated in the design of the study and coordination and helped to draft the manuscript. All authors read and approved the final manuscript.
